# Modernizing and harmonizing regulatory data requirements for genetically modified crops—perspectives from a workshop

**DOI:** 10.3389/fbioe.2024.1394704

**Published:** 2024-05-10

**Authors:** Nicholas P. Storer, Abigail R. Simmons, Jordan Sottosanto, Jennifer A. Anderson, Ming Hua Huang, Debbie Mahadeo, Carey A. Mathesius, Mitscheli Sanches da Rocha, Shuang Song, Ewa Urbanczyk-Wochniak

**Affiliations:** ^1^ Corteva™ Agriscience, Indianapolis, IN, United States; ^2^ CropLife International, Arlington, VA, United States; ^3^ BASF Corporation, Research Triangle Park, NC, United States; ^4^ Corteva™ Agriscience, Johnston, IA, United States; ^5^ Syngenta Seeds LLC, Research Triangle Park, NC, United States; ^6^ Bayer Crop Science, Chesterfield, MO, United States

**Keywords:** genetically modified (GM), regulation, food and feed, safety assessment, environmental risk assessment (ERA), problem formulation, cultivation, data requirements

## Abstract

Genetically modified (GM) crops that have been engineered to express transgenes have been in commercial use since 1995 and are annually grown on 200 million hectares globally. These crops have provided documented benefits to food security, rural economies, and the environment, with no substantiated case of food, feed, or environmental harm attributable to cultivation or consumption. Despite this extensive history of advantages and safety, the level of regulatory scrutiny has continually increased, placing undue burdens on regulators, developers, and society, while reinforcing consumer distrust of the technology. CropLife International held a workshop at the 16th International Society of Biosafety Research (ISBR) Symposium to examine the scientific basis for modernizing global regulatory frameworks for GM crops. Participants represented a spectrum of global stakeholders, including academic researchers, GM crop developers, regulatory consultants, and regulators. Concurrently examining the considerations of food and feed safety, along with environmental safety, for GM crops, the workshop presented recommendations for a core set of data that should always be considered, and supplementary (i.e., conditional) data that would be warranted only on a case-by-case basis to address specific plausible hypotheses of harm. Then, using a case-study involving a hypothetical GM maize event expressing two familiar traits (insect protection and herbicide tolerance), participants were asked to consider these recommendations and discuss if any additional data might be warranted to support a science-based risk assessment or for regulatory decision-making. The discussions during the workshop highlighted that the set of data to address the food, feed, and environmental safety of the hypothetical GM maize, in relation to a conventional comparator, could be modernized compared to current global regulatory requirements. If these scientific approaches to modernize data packages for GM crop regulation were adopted globally, GM crops could be commercialized in a more timely manner, thereby enabling development of more diverse GM traits to benefit growers, consumers, and the environment.

## 1 Introduction

Genetically modified (GM) crops that have been engineered to express transgenes have been commercially cultivated since 1995 and are annually grown on 200 million hectares globally. These crops have delivered important societal benefits, such as increased crop yields, resilience to adverse growing conditions, reduced tillage leading to improved soil health, reduction in the need for crop protection inputs, preservation of natural resources, and improved rural economies (Klümper and Qaim, 2014; Dively et al., 2018; Zilberman et al., 2018; Smyth, 2020; Ala-Kokko et al., 2021; Macall et al., 2021; Peshin et al., 2021; Brookes, 2022a; Brookes, 2022b; Brookes, 2022c). These benefits have led to rapid adoption of GM technology for agricultural production, including 80% of global cotton and 73% of global soybean. One-third of global maize production includes GM traits for herbicide tolerance, insect protection, or both (AgbioInvestor, 2023). GM traits have been introduced in other row crops such as oilseed rape, sugar beet, and alfalfa and, at a smaller scale, in specialty crops such as apples, eggplant, squash and potatoes (ISAAA, 2020). Hundreds of studies have been conducted to assess the safety of GM crops, and there have been no substantiated cases of resulting harm to people or livestock that consume GM crops or to the environment in which they are grown (European Commission, 2010; Snell et al., 2012; Van Eenennaam and Young, 2014; NASEM, 2016).

Despite this track record of safety and benefits, regulatory data requirements for approval and commercialization of GM crops have continued to grow globally. GM technology is primarily limited to major global crops, like maize and soybean, and to major input traits, such as insect protection and herbicide tolerance. While there are many efforts underway to use GM technology for other traits and to improve minor crops, especially for small holders in the developing world (David, 2009; Shelton, 2021; Woodruff, 2024), securing the regulatory approvals to enable cultivation and avoid potential trade disruptions can present often insurmountable challenges to commercialization. Only a few large multinational developers can afford the US$115 million cost and also persist for the 16 years that it currently takes, on average, to bring a new trait to the global market. More than one-third of those costs, and more than one-half of that time, are taken by the regulatory process (AgbioInvestor, 2022). These extensive and complex regulatory systems also mean that governments must invest significant resources in developing and maintaining regulatory bodies staffed with sufficient people and expertise, creating a burden on taxpayers and society. Countries that cannot afford such an investment are missing out on the benefits of GM crops.

**FIGURE 1:**
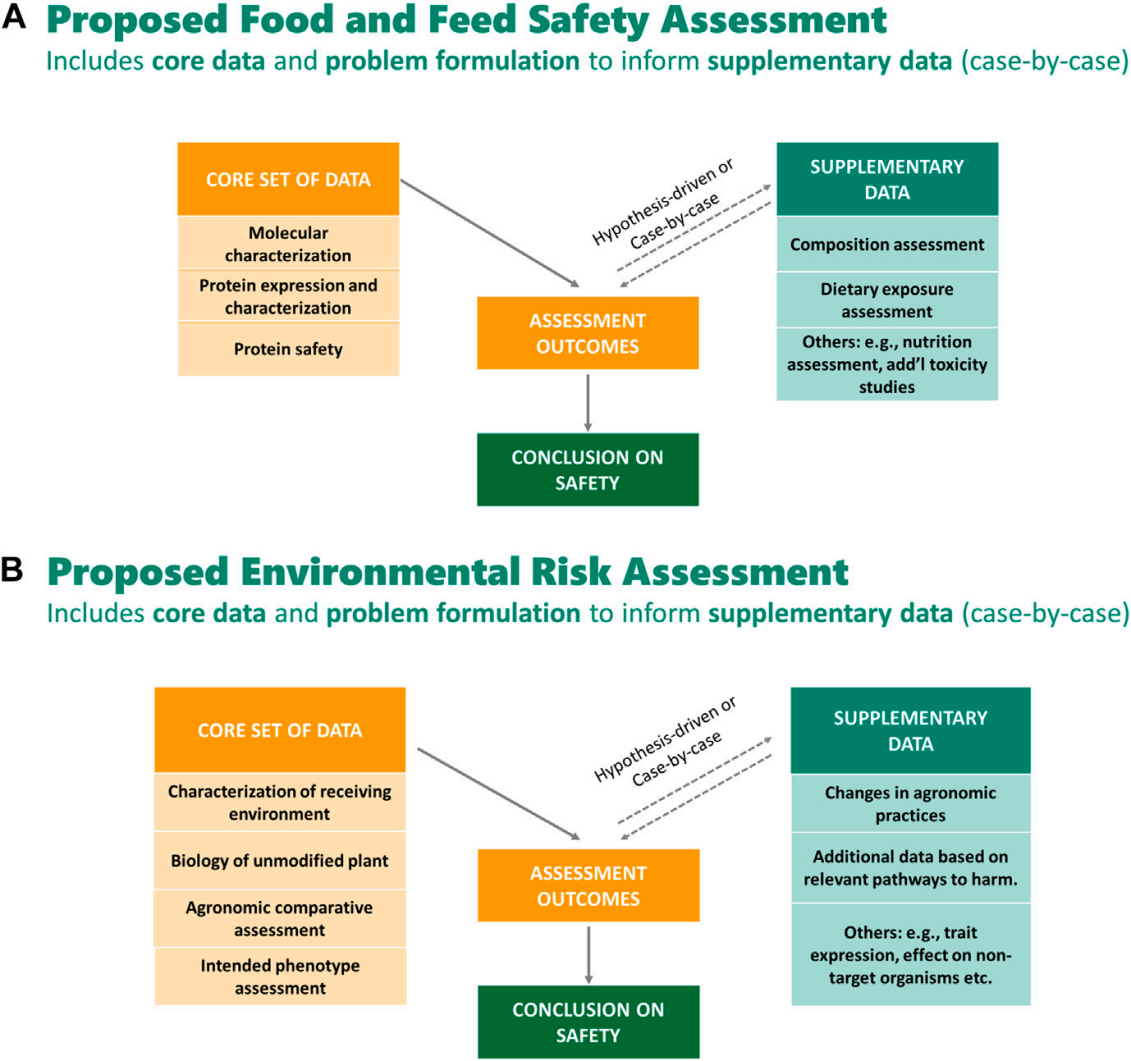
**(A)** proposes a set of data recommended for a science-based food and feed safety assessment for a typical GM crop and considers as core studies: basic molecular characterization, protein characterization and expression, and protein safety (i.e., history of safe use of the protein and source organism and bioinformatics to identify potential toxins and allergens). The outcomes of these core data are used to inform the problem formulation step and decide, on a case-by-case basis which, if any, supplementary studies are needed to make a conclusion on safety (Brune et al., 2021; Waters et al., 2021). **(A)** is adapted from Brune et al., 2021 and Waters et al., 2021. **(B)** proposes a set of data recommended for a science-based environmental risk assessment for a typical GM crop and considers as data: understanding the receiving environment and the basic biology of the unmodified plant; assessing the agronomic similarity of the GM crop to its conventional counterparts (i.e., agronomic comparative assessment); and understanding the intended trait of the GM plant and assessment of how the intended trait may lead to environmental harm. The core data should be used first to inform the problem formulation. If a conclusion cannot be made about the pathway to harm using the core data, additional case-by-case hypothesis-driven supplementary studies should be considered (Anderson et al., 2021).

CropLife International and its member companies that develop GM crops (BASF, Bayer Crop Science, Corteva™ Agriscience, and Syngenta) have proposed a modernized regulatory framework and streamlining of data requirements for GM crops that is based on scientific rationale and builds on the 25 years of experience with the technology, and the history of its safe use (Mathesius et al., 2020; Anderson et al., 2021; Bachman et al., 2021; Brune et al., 2021; Goodwin et al., 2021; McClain et al., 2021; Roper et al., 2021; Waters et al., 2021). The development of the proposed framework was motivated and guided by considering four key questions. 1) Are today’s regulations for GM crop approvals risk-proportionate? 2) Do today’s data requirements act as an unnecessary barrier to beneficial innovation? 3) How can knowledge and experience accumulated over the last 25 years inform modernization of regulations? 4) Can data requirements be streamlined and harmonized across countries and authorities? These questions were used to guide the determination of the types of data that are necessary to ensure GM crops are developed and deployed without increased risks for food and feed safety or the environment compared to conventional crops. Under this framework, core data, which are important for the problem formulation step of the risk assessment of the GM crop, were identified. The core data are used for problem formulation to identify plausible cause-and-effect hypotheses of harm from the GM crop. Depending upon the outcome of the problem formulation for a specific crop by trait combination, additional supplementary (i.e., conditional) studies may be needed, on a case-by-case basis, to analyze any plausible risk identified. Figure 1A outlines proposed core and supplemental studies for a Food and Feed Safety Assessment; Figure 1B outlines proposed core and supplemental studies for an Environmental Risk Assessment. CropLife International took an approach that is consistent with principles of risk assessment such that the proposed data requirements can fully inform decision-making by a regulatory agency, without the extraneous data present in many current regulatory submissions that does not meaningfully contribute to the risk assessment of the GM crop.

To further examine whether CropLife International’s proposed modernized data requirements are sufficient for food and feed safety assessments and for environmental risk assessments, a workshop was held at the 16th International Society of Biosafety Research (ISBR) Symposium (St. Louis, USA) in 2023. Using a case study of a hypothetical GM maize event containing two familiar transgenic traits (herbicide resistance and insect protection). The workshop participants were charged with considering whether the proposed data in the case study are scientifically both necessary and sufficient to determine the food, feed and environmental safety of the hypothetical GM crop.: CropLife International member representatives that served as moderators during the workshop authored this publication to report the outcomes and summarize the discussions that took place among the participants. The participants varied in their backgrounds and prior experience with risk assessment and included individuals from regulatory agencies, technology developers, consultant groups, and academia. A wide range of geographical areas were represented.

## 2 Case study description

For the case study, a hypothetical GM maize event was presented to the workshop participants for evaluation. The hypothetical event was intentionally simple for this exercise (i.e., a familiar crop with traits that are similar to many transgenic events that have already been reviewed and approved by regulatory agencies globally, with several in commercial production for many years), which enabled the participants to analyze in greater depth the need for data that is routinely submitted but may not contribute to the safety assessment. More specifically, a maize (*Zea mays*) event containing a single insertion encoding for two proteins from a single T-DNA introduced using standard disarmed Agrobacterium tumefaciens-based transformation was described. The two hypothetical traits provide protection against lepidopteran pests and tolerance to treatment with glyphosate herbicide, using a hypothetical Cry1 protein from *Bacillus thuringiensis* (*Bt*) and a hypothetical EPSPS protein variant isolated from maize, respectively. The workshop participants were asked to separately consider a food and feed safety assessment or an environmental risk assessment for this same hypothetical GM maize event. Additional distinctions between the presentation of the case study for the different assessments are outlined below.

### 2.1 Food and feed safety assessment

For the Food and Feed Safety Assessment, the results from hypothetical evaluations of core data on the characterization and safety assessment of the event were provided (summarized in Table 1). Throughout this paper, the term ‘data’ refers to both the results of experiments or studies as well as information gathered from literature reviews, consensus documents and other similar sources. As described in Waters et al. (2021), the core data for a food and feed safety assessment are: 1) molecular characterization, 2) protein characterization, and 3) protein safety (allergenicity and toxicity). The results of the molecular characterization demonstrated that there was an insertion of a single T-DNA sequence into the maize genomic DNA without any vector backbone sequences. There were no changes in the intended protein coding sequence and constitutive expression of both proteins were driven by familiar promoter elements (35S from cauliflower mosaic virus and ubiquitin promoter from *Zea mays*, respectively). Finally, the inserted DNA and the traits were indicated as being stable over three generations. The protein characterization data given to participants indicated that the molecular weight and amino acid sequence were as expected for both proteins. The function of the hypothetical Cry1 protein was established as having activity limited to target lepidopteran pest species, with no activity against other insect orders. Field tolerance to glyphosate from the hypothetical EPSPS protein variant was also as expected. The protein safety data indicated that both proteins are similar to proteins that have a history of safe use for food and feed; neither EPSPS proteins nor Cry proteins have any known toxicity or allergenicity concerns. Bioinformatics analysis comparing the amino acid sequences of both hypothetical proteins to a protein database also demonstrated that neither protein is related to any protein of toxicological concern nor related to any allergens in the qualified allergen database.

**TABLE 1:** Summary of food and feed safety assessment core data of the hypothetical GM maize.

Molecular characterization	
Number of insertion loci and inserts per locus	Insertion of one T-DNA from plasmid at a single locus. Based on sequencing of genomic DNA.
Presence or absence of unintended sequences (e.g., plasmid backbone)	Confirmed absence of backbone sequences from the transformation plasmid
Sequence of the inserted DNA and flanking borders	No changes in protein coding sequences. Small changes detected at junctions with genomic DNA.
Stability of the inserted DNA across multiple generations	Single T-DNA insertion is stably inherited over three breeding generations
Protein characterization and expression	
Identity of newly expressed proteins confirmed	EPSPS protein isolated from GM maize consistent with the theoretical molecular weight/amino acid sequence and the protein displayed expected enzyme activity
	Cry1 protein isolated from GM maize consistent with the theoretical molecular weight/amino acid sequence and the protein demonstrated expected insecticidal activity towards target insect pests
Protein expression as intended	EPSPS protein: Constitutive expression driven by ubiquitin gene promoter from *Zea mays*
	Cry1 protein: Constitutive expression driven by 35S promoter from Cauliflower mosaic virus
Protein safety	
History of safe use	EPSPS protein: History of safe use of source organism (maize) and similar EPSPS proteins
	Cry1 protein: History of safe use of source organism (*Bt*) and similar Cry1 proteins
Toxicity	Neither protein is related to any proteins of toxicological concern by bioinformatics search
Allergenicity	Neither protein is related to allergens in qualified allergen database by bioinformatics search

A familiar crop with familiar traits and minimal genetic disruptions was used for the workshop to promote discussion of what data is really needed to establish the food and feed safety of a GM crop event. It was also noted to workshop participants that extensive protein expression data in the plant was not obtained, nor was detailed proximate or nutrient composition data included. Further, while it was established that bioinformatics confirmed no homology to known allergens or toxins, no exposure assessments, no animal feeding studies, or other more direct assessments of potential for harm from the hypothetical event were included. As presented, the case study stated that considering 1) the assessment from the core data, 2) the familiarity of the crop and traits, and 3) the lack of direct interaction with other metabolic pathways of the plant, there was no hypothesis of food and/or feed safety risks for the new GM maize crop, and therefore additional supplementary data are not warranted to establish food and feed safety, in accordance with the approach established in Brune et al. (2021), McClain et al. (2021) and Roper et al. (2021).

### 2.2 Environmental risk assessment

For Environmental Risk Assessment (ERA), the intention of the case study was to model how problem formulation and core data should be leveraged to inform ERA of a GM crop for cultivation safety. Problem formulation is a process used in the ERA to develop plausible pathways to harm resulting from cultivation of the GM crop. Problem formulation first considers core data, then considers other data on a case-by-case basis if it is deemed necessary to inform the risk assessment. For ERA, core data includes information related to the receiving environment, description of basic biology of the unmodified plant, assessment of the agronomic similarity of the GM crop to its conventional counterparts, and characterization of the intended traits of the GM crop (summarized in Table 2). For the purpose of the case study, the protection goal was broadly stated as protection of biodiversity, specifically protection of beneficial or charismatic species. For the purposes of the workshop, the core characteristics of the event as described for the food and feed assessment were considered the same (e.g., molecular features), with additional information focused on agronomic and environmental aspects provided to guide the ERA discussion.

**TABLE 2:** Summary of environmental risk assessment core data.

Characterization of the receiving environment	
Receiving environment	Agroecosystem where Zea mays (maize) will be cultivated
Presence of wild relatives	There are no wild relatives of maize present in the targeted cultivation country
Changes in agronomic practices	There are no changes to the standard agronomic practices for hypothetical GM maize, relative to nonmodified maize
Description of the biology of unmodified *Zea mays*	
Survival	Maize requires human intervention for propagation and survival (OECD, 2003)
Weediness	Maize does not have weedy characteristics. While volunteers can occur the following season, maize is frost intolerant, the seeds have limited dispersal ability and they are not dormant (OECD, 2003)
Reproduction and gene flow	Maize propagates through seed and is wind-pollinated (OECD, 2003)
Agronomic similarity of the GM crop	
Multi-location field trial	A field study was planted during the 2022 growing season at 10 sites in the United States and Canada, which were selected to represent North American growing regions for commercial maize. Standard agronomic endpoints were assessed for hypothetical GM maize and nonmodified varieties. Results from this study demonstrate that hypothetical GM maize is agronomically similar to non-modified maize
Characterization of the intended phenotype	
Protein function	Hypothetical Cry1 protein - provides protection against lepidopteran insect pests: European corn borer (ECB), Asian corn borer, southwestern corn borer (SWCB), corn earworm (CEW), and fall armyworm (FAW)
	Hypothetical EPSPS protein- Functions in the chloroplast as a step in the biosynthesis of aromatic amino acids. EPSPS catalyzes the reversible reaction of shikimate-3-phosphate and phosphoenolpyruvate to produce 5-enolpyruvylshikimate-3-phosphate and phosphate. The EPSPS enzyme also serves as a selectable marker for plant transformation
Mode of action	Hypothetical Cry1 protein - ingestion of Cry1 is followed by receptor-binding in the insect mid-gut, which results in pore formation in the mid-gut of sensitive insects. The mode of action of Cry proteins in GM crops is well-documented (OECD, 2007)
	Hypothetical EPSPS - not inhibited by glyphosate and retains the standard EPSPS enzymatic function in the presence of glyphosate
History of safe use	Hypothetical Cry1 protein - History of safe use: Multiple crops have been globally assessed and approved as products that express Cry1 proteins (ISAAA, 2023)
	Hypothetical EPSPS - Multiple crops have been globally assessed and approved as products that express glyphosate-tolerant versions of EPSPS proteins from different sources (ISAAA, 2023)

The participants were presented with the following set of core data (summarized in Table 2) and were asked to consider if a plausible pathway to harm could be developed related to weediness, invasiveness, gene flow to wild relatives or hazard to non-target organisms: 1) assessment of the receiving environment indicating no wild relatives of maize present in the cultivation country and no changes to the standard agronomic practices relative to non-modified maize; 2) assessment of the basic biology of maize, using consensus documents, demonstrating non-modified maize has no weediness characteristics and requires human intervention for propagation and survival; 3) multilocation field trial data demonstrating hypothetical maize was agronomically similar to non-modified maize; and 4) assessment of the intended phenotype (i.e., insect protection and herbicide tolerant traits are not intended to increase fitness or survival in the environment).

Based on the core data assessed, the case study proposed that there are no plausible hypotheses for how cultivation of the hypothetical maize could result in environmental harm related to weediness, invasiveness, and gene flow to wild relatives. Thus, additional data will not further contribute to meaningful assessment of environmental safety. However, the case study proposed that a plausible pathway to harm to non-target organisms could be developed based on the intended insect protection phenotype. The hypothetical Cry1 protein was presented as providing protection against specific lepidopteran insect pests (European corn borer, Asian corn borer, Southwestern corn borer, corn earworm, and fall armyworm).

The mode of action of Cry proteins in GM crops is well-documented (Bravo et al., 2007; OECD, 2007). In this case study, additional supplemental protein expression data and non-target organism hazard data were provided to the participants, and they were asked to consider if additional plausible pathways to harm could be developed. The set of supplemental data (summarized in Table 3) was as follows: 1) multilocation field trial data measuring the concentration of the hypothetical Cry1 protein in several plant tissues to inform exposure assessment; 2) an exposure assessment for different non-target organisms to consider the likelihood and magnitude of exposure to the hypothetical Cry1 protein; and 3) results of non-target organism Tier I hazard studies for several surrogate species representing different taxonomic orders (e.g., ladybird beetle, a soil dwelling organism, and a non-target predator) conducted with the Cry1 protein in the diet.

**TABLE 3:** Summary of environmental risk assessment supplementary data.

Expression of the hypothetical Cry1 protein in GM maize	
Multi-location field trial	• A field study was planted during the 2022 growing season at 10 sites in the United States and Canada, which were selected to represent North American growing regions for commercial maize. Hypothetical Cry1 protein expression was analyzed from representative plants at 6 sites • Hypothetical Cry1 protein expression in GM maize was measured in several plant tissues, including pollen (R1), leaf, root, and whole plant (several vegetative and reproductive growth stages) • Results from this study: the hypothetical Cry1 protein in GM maize is below the limit of detection (LOD) of the analytical assay in pollen and root. The hypothetical Cry1 protein in GM maize was detectable in leaf and whole plant, with the highest concentration detected in R1 leaf (mean = 30 ng/mg; maximum = 45 ng/mg)
Specificity	
Specificity of the Cry1 protein	• Cry1 protein activity is well-documented to be limited to the order Lepidoptera (Van Frankenhuyzen, 2009; Anderson et al., 2021) • The hypothetical Cry1 protein provides protection against lepidopteran insect pests, including European corn borer (ECB), Asian corn borer, southwestern corn borer (SWCB), corn earworm (CEW), and fall armyworm (FAW)
Exposure assessment—non-target organisms	
Ladybird beetle	• May consume pollen, plant tissues, or prey that have previously consumed plant tissues • For the purposes of this case study, a worst-case scenario would assume a ladybird beetle consumes GM maize leaf tissue
Soil dwelling organism	• Detritivores may consume roots or plant tissues that have fallen to the ground • There is no exposure to detritivores via roots (root hypothetical Cry1 protein expression is below LOD) • For the purposes of this case study, a worst-case scenario would assume a detritivore consumes GM maize leaf tissue
Aquatic organism	Although aquatic habitats may be located near agricultural areas, exposure of aquatic organisms to biotech crops is limited temporally and spatially (Bachman et al., 2021) and aquatic exposure to Bt corn is extremely small (US-EPA, 2010)
Non-target predator	A non-target predator may consume prey that has previously consumed the hypothetical GM maize plant tissues. For the purposes of this case study, a worst-case scenario would assume there is no degradation of the hypothetical Cry1 protein in the prey; however, previously it has been shown prey contains lower concentrations of Cry protein relative to the Cry protein concentration in planta (Raybould et al., 2007)
Non-target honey bee	There is no exposure to honeybees (pollen hypothetical Cry1protein expression is below LOD). Non-target lepidopteran–non-target Lepidoptera do not consume maize pollen directly, but they may ingest maize pollen that has been deposited on host plants growing within or closely adjacent to maize fields. There is no exposure to pollen-feeding non-target lepidopterans (expression in pollen is below LOD)
Hazard assessment—non-target organisms	
Ladybird beetle	Tier I study was conducted; diet contained hypothetical Cry1 protein at approximately 10x the environmentally relevant exposure. The no observable adverse effect concentration (NOEC) was >10X the environmentally relevant exposure, resulting in a margin of exposure >10
Soil dwelling organism	Tier I study was conducted; diet contained hypothetical Cry1protein at approximately 10x the environmentally relevant exposure. The no observable adverse effect concentration (NOEC) was >10X the environmentally relevant exposure, resulting in a margin of exposure >10
Aquatic organism	Tier I study was not conducted because aquatic exposure to Bt corn is extremely small (US-EPA, 2010)
Non-target predator	Tier I study was conducted; diet contained hypothetical Cry1protein at approximately 10x the environmentally relevant exposure. The no observable adverse effect concentration (NOEC) was >10X the environmentally relevant exposure, resulting in a margin of exposure >10
Non-target honeybee	Tier I study was not conducted because there is no exposure to honeybee (expression in pollen is below LOD)
Non-target lepidopteran	Tier I study was not conducted because there is no exposure to pollen-feeding non-target lepidopterans (pollen hypothetical Cry1 protein expression is below LOD). Lepidoptera that consume maize leaf tissue or grain are considered maize pests
Fate of the hypothetical Cry1 protein in the environment	
Soil Fate	There is a large body of evidence that Bt Cry proteins do not accumulate or persist in soil (Clark et al., 2005; Stotzky, 2005; Icoz and Stotzky, 2008)

The multilocation field trial data showed that the Cry1 protein was only detectable (above the limit of detection) in the leaf and whole plant, with the highest concentration found in R1 leaf. The protein was below the limit of detection of the analytical assay in pollen and root. Based on the tissue expression, the exposure assessment concluded that since there is no expression of the Cry1 protein in pollen, there would be no route of exposure to non-target pollen feeding organisms (e.g., honeybee). Finally, the Tier I hazard studies indicated that no hazard was observed at concentrations that exceeded >10x the expected environmental concentration.

Usually, the assessment of adverse effects in non-target organisms follows a tiered approach that starts with laboratory studies at levels that exceed worst-case exposure conditions (Romeis et al., 2011). Tier I laboratory studies with non-target organisms are typically conducted using at least 10X the worst-case expected environmental concentration. In this case, the results of the hypothetical Tier I dietary studies indicated no hazard (i.e., adverse effects) at concentrations that exceeded 10x the worst-case expected environmental concentration, and thus a conclusion that evidence is sufficient without conducting additional hazard testing was indicated. Based on data from the exposure assessment and non-target hazard assessment studies, the case study proposed that there were no plausible pathways to harm to non-target organisms due to lack of exposure and/or lack of risk because there were no adverse effects at concentrations that exceeded 10X the worst-case expected environmental concentration. Participants were asked to consider whether they agreed with the conclusions proposed by the case study based on core data and additional supplementary data related to protein expression, non-target organism exposure, and non-target organism hazard.

Additional information such as molecular data to confirm that the insert is an intact single copy, stable across generations, and that there is no insertion of DNA from the plasmid backbone were not provided in the ERA case study. These additional data for product characterization have historically been submitted to regulators as part of cultivation applications, but they are not directly relevant to ERA (Anderson et al., 2021).

## 3 Learnings from breakout group discussions

After participants attended the introductory presentation session of the workshop, they were distributed into smaller discussion groups of approximately 10 people, with CropLife International member representatives serving as moderators. Each participant had the opportunity to choose either the Food and Feed Safety Assessment or the Environmental Risk Assessment, depending on their respective areas of interest.

The goal of the smaller group discussion sessions was to allow participants to go into deeper conversations about the proposed modernized paradigm for a risk assessment of a GM crop. Discussions were aided by a distribution of a printed booklet that included a description of the hypothetical GM maize event and the data collected, and that outlined the key concepts of using the core data for a Food and Feed Safety Assessment and Environmental Risk Assessment. Moderators provided some time for the participants to review the information and then introduced the case study by giving a brief overview of the information provided in each data section of the case study. Participants were encouraged to provide feedback and to bring up questions and/or comments about topics/elements of the case study that they considered not sufficiently covered by the data provided. They were also asked to complete a worksheet allowing for comments on the specific steps of the assessment process.

Discussions during this small group session were productive and highly informative. Overall, the participants were engaged, willing to discuss, and mostly supportive of the general assessment framework of primarily using core data and only using further assessments on a case-by-case basis.

A summary of key points from the breakout group discussions is shared below. This section is not intended to be a complete summary of the discussion, rather the authors have captured points of interest with an emphasis on points that are worth considering for future workshops and discussions on this topic.

### 3.1 Food and feed safety assessment

In the small group session, participants were asked to consider 1) the assessment from the core studies (see Table 1), 2) the familiarity of the crop and traits, and 3) the lack of direct interaction with other metabolic pathways of the plant, and then decide whether there was a hypothesis of food and/or feed safety risks for the new GM maize crop. Because of these considerations, the position for the case-study was that, for the hypothetical event, additional supplemental studies are not warranted to establish food and feed safety, and the participants discussed whether they agreed with this position.

Below are some key feedback and questions captured during the workshop regarding the proposed approach for the assessment of Food and Feed Safety of the hypothetical GM maize event.

#### 3.1.1 Molecular characterization (transformation method, transformation construct, DNA insert characterization)

Overall, the participants agreed that the proposed molecular characterization core data is aligned with what is currently provided and that the information was sufficient to inform a food and feed safety assessment. One potential exception to the core data package that was discussed is data demonstrating that the insert is stable over at least three generations. The participants suggested that this study could be considered as supplemental, and not necessarily required as part of the core data package, if the insert is demonstrated to be inserted into the chromosome and is not interrupting endogenous genes or regulatory elements, and there is no other reason to expect that the insert might be unstable (e.g., insertion site near a transposon). There was some discussion that three generations of data may not be considered enough by all regulatory agencies and that additional generations could be required for polyploid crop species. Additionally, participants raised questions about Agrobacterium transformation not being targeted and discussed providing data on whether any internal genes were modified. It was also noted by workshop participants that the use of Next-Generation Sequencing (NGS) to characterize the insert is not yet accepted by all regulatory agencies, but also there was recognition of the utility of NGS to provide a more comprehensive characterization of the insert and the insertion site compared to traditional methods (e.g., Southern blots).

#### 3.1.2 Protein characterization (molecular weight, protein sequence confirmation, protein function)

Participants agreed that the protein characterization information was sufficient to inform the food and feed safety assessment, with some discussions around whether a registrant would always be able to provide what is required, as some proteins may be more challenging to characterize (e.g., difficulties in isolating the proteins in an active form, generating specific antibodies, or generating SDS-PAGE and Western blot data). A question was also raised on maize codon optimization and if the protein would still be considered the same as the native version. Future workshops can reinforce that maize codon optimization of the GM trait gene does not alter the trait protein sequence. Thus, it should not change the safety profile of the protein if there is no change to the amino acid sequence. Discussion also occurred regarding familiarity with promoters and the relationship to expression levels. The participants discussed if there might be a need to better understand the protein expression levels for unfamiliar promoters and also if increased expression levels might raise a concern of potentially increased allergenicity risk.

#### 3.1.3 Protein safety/toxicology/allergenicity (background, source, history of safe use, bioinformatics)

Participants agreed that the EPSPS protein information for safety was sufficient to inform the food and feed risk assessments, but questions were raised about Cry proteins around digestibility and heat stability. There was also discussion regarding how similar a protein would need to be to a known protein to be considered familiar. Additionally, concerns were raised in the small group discussion on the limited protein expression data provided in the case study as it related to an exposure assessment. In response, the moderators noted that an exposure assessment is not necessary, because no hazard was identified from the proteins. However, when a hazard is identified, then protein expression levels are needed to enable assessment of potential exposure (Brune et al., 2021).

#### 3.1.4 Additional information needed to determine event safety

It was stated by one participant that if there was a disruption of a native gene, then composition data could be requested. Discussion also occurred regarding the concept of History of Safe Use (HOSU), and the amount of data, time and similarity (e.g., consideration of minor protein sequence differences) needed to establish something as having sufficient familiarity to be considered safe without additional data. One participant suggested that protein sequence data would be needed to demonstrate a HOSU and could be useful in determining the activity of the protein.

#### 3.1.5 General feedback for food and feed safety assessment

Although participants generally agreed that the case study with a familiar crop and familiar traits is a good starting point for the discussions, several suggestions were made for further discussions to also provide a case study on an unfamiliar event or protein, to lay out how each study informs the safety assessment, to provide more on the problem formulation process, and to provide more graphics and to use examples. Discussion also occurred around the challenges of communicating and making changes to the currently provided data in regulatory applications. On this topic, proposals from participants included suggestions to emphasize more the end goal of getting needed products on the market sooner with less regulatory burden for all stakeholders and to publish more data prior to submission of the application in the scientific literature, and to be ready to provide additional data upon request.

### 3.2 Environmental risk assessment

After introducing the case study, the CropLife International moderator described a list (provided with the case study) of the specific potential pathways to harm that are relevant to the cultivation of the hypothetical maize event. Additionally, an explanation for how the core data can be used to sufficiently assess environmental risk was provided. For plausible pathways to harm that may not be sufficiently addressed by the core data (i.e., potential harm to non-target organisms), another list of potential pathways to harm that are specific to non-target organism (NTO) exposure was also presented.

Below are some key feedback and questions captured during the workshop regarding the proposed approach for the Environmental Risk Assessment of the hypothetical GM maize event.

#### 3.2.1 Weediness potential

There was an overall consensus among the workshop groups that weediness can be adequately assessed using only core data. Participants agreed that there is not a plausible pathway to harm in the case study since maize is highly domesticated and volunteers will not survive without human intervention and management. One group discussed questions around the potential for dormancy, which may be a weediness trait, and whether it can be assessed in the core data (multilocation field trial; Table 2). It was concluded within the groups that the similarity in agronomic characteristics between the GM maize event and the non-GM maize in the case study core data is sufficient to show that there is a highly unlikely risk of weediness potential. This follows the principle of placing risk in the context of current practice (i.e., that the modified maize will have no greater risk than that of cultivation of the non-modified maize) (Raybould and MacDonald, 2018). However, one workshop group had unresolved discussions on whether a difference in agronomic performance between different geographical regions may result in differences in the risk assessment and what specific agronomic elements are the most relevant to consider. Some participants in this group proposed scenarios in which the agronomic data generated in field trials performed outside of the cultivation country may not sufficiently represent the agronomic outcomes of field trials performed within the cultivation country.

#### 3.2.2 Gene flow potential to wild relatives

There was general consensus that there is no environmental safety concern of gene flow in the case study based on the core data because there were no wild relatives present in the hypothetical cultivating environment. There was some interest from participants in further exploring how the risk assessment and data requirements will change if the cultivation environment did contain wild relatives. Also, there was some discussion on the threshold of relatedness between the GM maize and a wild relative species that constitutes a safety concern in terms of gene flow. Ultimately, there was additional consensus that product registrants should demonstrate that there are no wild relative species that are reproductively compatible with GM maize (regardless of species relatedness) to position that there is no gene flow concern. Alternatively, if there are wild relative species in the area of cultivation an assessment of the likelihood and consequences of trait introgression into the wild relative population may be warranted based on a problem formulation approach (Anderson et al., 2021). Participants generally stressed the importance of citing published literature (e.g., accepted consensus references on crop-specific biology) as part of the core data to support the environmental risk assessment. Although it was acknowledged that gene flow will not likely occur between GM maize and wild relatives in the case study example, there was some discussion around whether gene flow may occur between the GM maize and adjacent local non-GM maize varieties and negatively impact crop integrity and biodiversity. The case study focused on assessing plausible pathways to harm related to gene flow between GM maize and sexually compatible weedy relatives. Future workshops can address concerns that were raised about coexistence of GM and non-GM cropping systems. Such a workshop may have to distinguish between environmental risks and market or socio-political concerns. For example, countries that have landrace populations for which the genetic make-up *per se* is a protection goal may have societal concerns about coexistence (for example, there could be changes the genetic identity of the landrace).

#### 3.2.3 Plausible pathways to harm for non-target organisms (NTO)

All groups aligned that the only plausible pathway to harm from the case study that could not be sufficiently addressed with core data alone was the potential for harm to NTOs from potential exposure to the hypothetical Cry1 protein (Table 2). Participants discussed the plausible pathways to harm that are specific to NTOs. There was general agreement that no additional data was needed to assess the potential for the EPSPS protein conferring the herbicide tolerance trait to cause harm to NTOs. However, participants acknowledged that public perception of herbicide tolerance traits could influence regulatory decisions and may need to be considered when determining the registrability of a GM crop. Such perceptions are not reflective of an actual risk, and the additional data generated do not inform the science-based risk assessment. For other pathways to harm, there was consensus that if there was either no hazard or no detectable exposure, then there is low risk to NTOs. For example, honeybees that may directly consume maize pollen and NTO lepidopterans that may indirectly consume maize pollen that drifts onto their host plants should have low risk in the ERA case study since the GM maize event has expression less than the limit of detection (LOD) of the insecticidal protein in pollen tissue (Table 2). It was generally accepted by workshop participants that if expression of the insecticidal protein is <LOD in tissues that might be consumed by an NTO, further toxicity testing to determine hazard is not warranted.

Participants were also mostly aligned that aquatic environments generally experience minimal exposure to GM crop tissue and so additional toxicity testing is not needed for aquatic NTO species in most situations. However, some participants expressed uncertainty on whether this may be an issue if GM crops are cultivated very close to aquatic environments, which may affect exposure levels to NTO aquatic species. For NTO species where there is a plausible pathway to harm, all groups agreed that further data (exposure assessment or NTO Tier I laboratory testing) might be needed. Some discussions among participants regarding appropriate surrogate species to use for NTO testing and to what extent test species need to match those found in the cultivation regions were not resolved in the workshop. There was some additional discussion around the large body of scientific literature describing the surrogate species concept for testing Cry proteins and other types of plant incorporated protectants (e.g., Romeis, et al., 2011; Romeis et al., 2013; Bachman et al., 2021). While the terms “focal species” and “indicator species” were not discussed directly as part of the workshop, understanding protection goals and selecting appropriate surrogate species or indicator species to inform the science-based assessment of risk is an important consideration (Rose, 2007; Roberts et al., 2020). Despite the lack of consensus on species selection, there was clear alignment among participants that NTO species representatives should only be tested if there is a valid hypothesis that there is a plausible pathway to harm for that specific organism type. For this reason, NTO studies should only be conducted when hypothesis-driven (Figure 1B).

#### 3.2.4 General feedback and future considerations for ERA

Although participants agreed that a generic ERA case study is a good starting place, participants indicated that future workshops using a modified case study tailored for specific geographical regions will be even more helpful. As different countries have different sets of questions and concerns from local regulatory agencies, using more country-specific scenarios and less familiar pest-control traits in a case study may be more directly relevant in that region.

Related to gene flow, there was not a consensus about potential for harm in small team discussions. Future workshops would benefit from guided discussion to help develop problem formulation for gene flow. For example, it could be established as a baseline that for gene flow to occur naturally in the environment, and when assessing the potential for harm from gene flow between GM maize and local maize varieties, it should be compared to potential for harm from gene flow of non-GM maize and local maize varieties (OECD, 2023). Furthermore, future workshops can reinforce that if gene flow to local maize varieties is a relevant concern for a specific cultivation country, then there is a large body of literature to leverage to assess if additional data is needed to inform the risk assessment (See OECD, 2023 Annex B for recent review) such a workshop would need to distinguish between the true environmental impact and concerns related to trade or economic issues.

Also, there were productive discussions on the topic of data transportability. Participants generally accepted the concept of transportability for lab study data. However, due to a lack of time for discussion, some unresolved questions remained regarding the transportability of field study data. Future workshops will benefit from guided discussion to help explain the principle of data transportability. An underlying principle of data transportability is that if no biologically relevant differences between a GM crop and its conventional counterparts are observed in one country or region, data from these studies can be used to inform the risk assessment in another country, regardless of agroclimatic zone (Bachman et al., 2021). Following the recommendations for modernizing global regulatory frameworks for GM crops, additional agronomic data should only be collected in the local environment if there are plausible pathways for harm that cannot be fully informed by the core data.

Furthermore, there was some interest from participants in discussing how the proposed risk assessment paradigm might apply to combined GM products (i.e., breeding stacks), yield and stress traits (e.g., drought resistance), and streamlining of import registrations.

One topic that generated discussion across groups was the value of product characterization data in an environmental risk assessment. In the proposed modernized regulatory framework (Anderson et al., 2021), underlying characterization data for the GM event are not regarded as core to the regulatory assessments (such as molecular data to confirm that the insert is an intact single copy, stable across generations, and that there is no plasmid backbone DNA). Although these data do not directly inform the ERA (Anderson et al., 2021), it was discussed that an understanding of the characteristics of the GM product provides foundational information that enables the regulatory assessments to focus on the intended introduced trait during the problem formulation stage. Therefore, it was proposed to consider including, as part of the modernized ERA framework, a set of foundational information and data from the characterization of the GM event that confirms that (1) the intended gene sequence was inserted and functions as intended, as well as the number of such insertions; (2) the plants produce the intended newly expressed protein (NEP); (3) the intended phenotype is achieved.

## 4 Key considerations and takeaways from the workshop

The case study for the workshop considered a single event, albeit one that contained genetic material encoding for two proteins leading to two distinct traits (herbicide tolerance and insect protection). However, the majority of commercialized products contain multiple GM events that are combined through conventional breeding (also known as stacked trait products). The typical regulatory process first assesses all single events, before applying regulatory processes, if any, to the stacked trait products. In this sense, the case study used for the workshop reflected a realistic scenario in which regulators assess a single event regardless of whether the event will be commercialized as a single event or as a stacked trait product.

Regulatory processes for stacked trait products vary globally, with many countries recognizing the long, safe history of conventional breeding and not requiring additional assessment once all the single events are approved. It is the position of CropLife International that additional safety assessment of a stacked trait product produced by conventional breeding should not be required unless there is a plausible and testable hypothesis for interaction of the traits (Goodwin et al., 2021). This case study did not address stacked trait products however, further iterations could include consideration of stacked trait products and how to evaluate possible interaction of traits.

The workshop was convened to explore the proposed modernized data requirements for regulatory assessments of GM crops (Anderson et al., 2021; Waters et al., 2021). The participants were charged with considering whether currently implemented regulations for GM crops are risk-proportionate or whether they create an unwarranted barrier to the introduction of new traits. The organizers presented a position that knowledge and experience from 25 years of research and development could inform regulatory modernization and that streamlined data requirements could advance harmonization across countries and authorities.

Overall, considering the case study discussed, the participants at the workshop found the proposed modernized data requirements generally to be necessary and sufficient for decision making to support the safe commercial introduction of a new GM crop. There was a clear consensus that some of the current data requirements are no longer routinely warranted for familiar traits such as that discussed in the case study, given the track record of GM crops not presenting unexpected or unintended effects on food or feed safety or environmental risk relative to their conventional counterparts. Participants appreciated the benefit of harmonized hypothesis-based risk assessments to enable future deployment of GM crops that can address emerging agricultural challenges associated with increasing demand for affordable healthy food and changing agricultural environments. The points discussed in this publication will be used to further clarify recommendations for supplementary case-by-case data and guide the development of future, more targeted workshops and related discussions. In particular, applying the proposed framework to traits and crops with which there is less familiarity and established HOSU than those used in the case study may be associated with greater uncertainty in the foundational information of the GM event. Additional case studies involving less familiar traits and different crops should be used to further test the robustness of the modernized regulatory framework.

The workshop focused on what data was scientifically necessary and sufficient to make a conclusion on the food, feed and environmental safety of the GM crop. However, several participants noted that certain data not included in the case study was either required in their jurisdiction or routinely submitted by applicants. While it was beyond the scope of this workshop, future targeted workshops or symposia could address the extent to which regulatory authorities have the flexibility to decide, on a case-by-case basis, what data is necessary to make a conclusion on safety. In some jurisdictions the recommendations of the modernization project could be implemented by applicants by including a scientific rationale in their submission for why a specific study is not necessary. In other cases, changes to laws, regulations, or written guidance would be needed to implement these recommendations.

The case study for the first workshop, as described in this publication, was a valuable tool to foster discussion about science-based data requirements for the assessment of GM crops. If these scientific approaches to modernize data packages for GM crop regulation were adopted globally, delays to the commercialization of GM crops could be reduced, thereby allowing farmers access to new GM traits that will benefit not just growers, but consumers and the environment as well. For more information on the case study used in the workshop, or if there is interest in hosting a similar workshop, please contact the corresponding author.
